# In vitro dynamics of HIV-1 BF intersubtype recombinants genomic regions involved in the regulation of gene expression

**DOI:** 10.1186/1743-422X-6-107

**Published:** 2009-07-16

**Authors:** Mauricio G Carobene, Christian Rodríguez Rodrígues, Cristian A De Candia, Gabriela Turk, Horacio Salomón

**Affiliations:** 1 National Reference Center for AIDS, Department of Microbiology, School of Medicine, University of Buenos Aires, Buenos Aires, Argentina

## Abstract

HIV-1 intersubtype recombination is a very common phenomenon that has been shown to frequently affect different viral genomic regions. Vpr and Tat are viral proteins known to interact with viral promoter (LTR) during the replication cycle. This interaction is mainly involved in the regulation of viral gene expression, so, any structural changes in the LTR and/or these regulatory proteins may have an important impact on viral replication and spread. It has been reported that these genetic structures underwent recombination in BF variants widely spread in South America. To gain more insight of the consequences of the BF intersubtype recombination phenomenon on these different but functionally related genomic regions we designed and performed and *in vitro *study that allowed the detection and recovery of intersubtype recombinants sequences and its subsequent analysis. Our results indicate that recombination affects differentially these regions, showing evidence of a time-space relationship between the changes observed in the viral promoter and the ones observed in the Vpr/Tat coding region. This supports the idea of intersubtype recombination as a mechanism that promotes biological adaptation and compensates fitness variations.

## Background

Recombination among retroviral genomes was first documented in avian tumour viruses by Vogt et al in 1971 [1] and subsequently in other retroviruses [2,3]. This phenomenon occurs before integration at a high rate along the reverse transcription stage. It is dependent on co-packaging of two different viral genomes [4,5], and provides a powerful mechanism to rapidly increase viral sequence diversity [6-8].

It has now become evident that HIV recombination is a very common event and in areas with different circulating subtypes, recombinant viruses may even predominate. To date, more than 40 circulating recombinant forms (CRFs) have been described (Los Alamos HIV Database) reinforcing the idea that HIV-1 intersubtype recombination is a very effective way to augment variability and to improve viral fitness [9].

In previous studies, our results showed that the epidemic in Argentina is characterized by the high prevalence of a circulating recombinant form, CRF12_ BF and many related BF recombinant forms [10-13]. Molecular studies on these variants showed that recombination frequently affected genomic regions involved in regulating viral gene expression, replication, and interaction with the host immune system, eventually leading to remarkable functional consequences [14,15].

Transcriptional activation of HIV-1 gene expression is controlled in part by the interaction between cellular and viral transcription factors and the HIV-1 long terminal repeat sequences (LTR). Tat is a viral transactivator that activates HIV transcription through complex interactions with RNA and host cell factors, while Vpr, a small virion-associated protein, has been shown to play multiple functions in the viral replication cycle including transactivation of viral [16] and host cell genes, regulation of the reverse-transcription process accuracy, viral DNA nuclear import, cell cycle progression, and apoptosis regulation.

As stated above, Vpr and Tat are viral proteins known to interact with LTR and regulate gene expression. Then, variations in LTR and/or these regulatory proteins may have an important impact on viral replication and spread.

Thus, the aim of this study was to gain more insight of the consequences of the BF intersubtype recombination phenomenon on the different but functionally related genomic regions LTR and Vpr/Tat, through the development of an *in vitro *time-course experiment that allowed the detection and recovery of intersubtype recombinants sequences and its subsequent analysis.

## Results

### Detection of intersubtype recombinants

**Figure 1:**
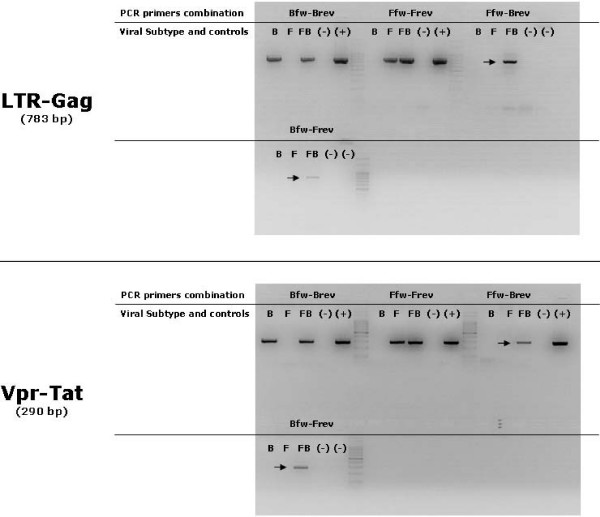
**Detection of BF intersubtype recombinant genomes by PCR amplification**. Proviral DNA found in samples from mono (B or F Subtype) and dual-infected (B+F) cultures was used to obtain four different amplicons from the genomics regions under study. Primers combinations and viral strains (B, F or FB) present in each cell culture are indicated (as described in Methods). Upper and lower panels show the result of the amplification of the LTR-Gag and Vpr/Tat regions, respectively. Intersubtype recombinant PCR products are indicated by arrows. Positive and negative controls were included in the reactions.

Intersubtype recombinant forms were first detected by PCR amplification (figure 1) of the Vpr/Tat and LTR-Gag regions at days 3 and 7, respectively. Although different amounts of template DNA were used, no LTR-Gag recombinants were detected before day 7. Recombinant PCR products from days 3, 7, 10 and 18 of the Vpr-Tat region, and from days 7, 10 and 18 of LTR-Gag region were cloned into a commercial vector and automatically sequenced. Both BF and FB primer combinations were used in each amplification reaction. A total of 61 Vpr-Tat and 68 LTR-Gag sequences were obtained, and an extensive molecular analysis was carried out.

### LTR and Vpr-Tat recombinant sequences analysis

Molecular analysis of the viral promoter sequences was focused on breakpoint patterns, and sequence changes in transcription factors binding sites and regions involved in RNA secondary structures.

LTR-Gag sequences showed two patterns of breakpoints distribution: pattern I was defined by a recombination breakpoint located in nucleotide position – 327 in the LTR modulatory region, while pattern II presented a recombination breakpoint in nucleotide position 790, just prior to the p17 Gag start codon (positions are based on HXB2 numbering). Regarding the latter, naturally occurring BF recombinants also frequently showed this recombination pattern (data not shown). As depicted in table 1, frequency of sequences showing pattern I or II changed over the time.

**Table 1:** Distribution and frequency of recombination patterns in the LTR/Gag sequences

**Day**	**Pattern I**	**Pattern II**	**Total**
**7**	9 (40.9%)	13 (59.1%)	22

**10**	7 (29.2%)	17 (70.8%)	24

**18**	-	22 (100%)	22

The pattern frequency analysis, performed in the context of the BF or FB recombinant nature of the sequences and sampling day (7, 10 and 18), showed that pattern II was always the most frequently found, even though the coexistence of pattern I was evident in samples from days 7 and 10. This was observed in both the BF or FB groups of sequences.

The TAR sequence, an important regulatory element positioned immediately after the transcription starting site (nt +1 to +59) also showed a high level of conservation between recombinant sequences and over time. A few sequences presented nucleotides changes located in the stem region. Two sequences from day 7, exhibited an A→G substitution at position +28, and one sequence from day 18 had G→A at position +27. Also 5 sequences, 1 from day 7, 1 from day 10 and 3 from day 18, presented an A→G substitution at position +15. Its important to highlight that this change has been documented before as present in the prototypic BF recombinant sequence CRF12_BF [14].

The dimerization initiation signal (DIS) is a 6 nt palindromic sequence located at the loop of the proposed stem-loop 1 (SL1) in the 5' untranslated region of the HIV-1 genome. It plays an important role in both RNA dimerization and RNA packaging. SL1 from the parental B and F subtypes only differ in their DIS hexanucleotide. The analysis showed a high conservation of SL1 in all the recombinant sequences. BF or FB recombinants conserved the intact F (GUGCAC) or B (GCGCGC) DIS sequence, respectively.

Overall, analysis of LTR variability in major regulatory sites was performed, including NFAT, C-ETS, Core NRE, TCF-1α, AP-1, RBF2, C/EBPI, NFκB (I, II), SP1 (I, II, II), TATA Box (CATATAA), and E-Box (USF-binding site). A high degree of conservation was observed in all of them but in NFκB and Sp1 sites.

The HIV-1 promoter contains 3 Sp1 and 2 NF-kB binding sites that regulate gene expression via the recruitment of both activating and repressing complexes.

**Figure 2:**
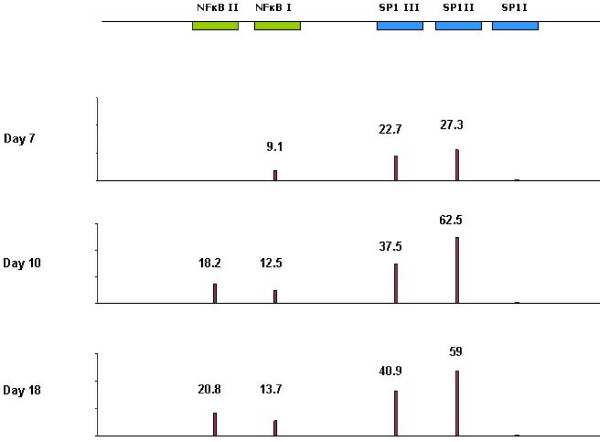
**Frequency of sequences harboring mutations in SpI and NF-κB binding sites over the time-course experiment**. Diagram depicts an schematic representation of the NF-κB and SpI sites distribution in the HIV-1 LTR U3 region and the frequency (expressed as percentages) of sequences presenting mutations in NF-κB or Sp1 binding sites in relation to each time point.

Analysis of the three Sp1 sites showed that Sp1I (GGGGAGTGGC) site was found to be conserved among all the recombinant sequences, and that Sp1II was the most frequently affected by nucleotide changes, followed by Sp1III. On day 7, 27.3% and 22.7% of the sequences harbored non-parental nucleotide changes at Sp1 II and Sp1III binding sites, respectively, changing to 62.5% and 37.5% at day 10, and to 59% and 40.9% at day 18 of the time-course experiment (Figure 2). Position 5 in the 10-bp Sp1 II site (TGGG**C**GGGAC) and positions 9 and 10 in the Sp1 III (GAGGCGTG**GC**) were the most frequently changed. Nucleotides were substituted for a T or an A.

Regarding NF-κB sites (GGGACTTTCC), also an increasing number of sequences presented mutations in both sites I and II over the time span (Figure 2). Nevertheless, site II was found to be conserved among the sequences from day 7. Nucleotide changes were observed in positions 9 and 10 (GGGACTTT**CC**), and 5 and 10 (GGG**A**CTTTC**C**) of the NF-κB II and NF-κB I sites, respectively.

In contrast with the observed for the viral promoter sequences, Vpr/Tat sequences displayed a less restricted recombination breakpoints distribution. Considering this, sequence analysis was performed taking into account if the breakpoint was located in the Vpr or in the Tat coding region from a total of 61 sequences, 63.9% (n = 39) showed recombination breakpoints in the Vpr coding region (Pattern Vpr), and 36.1% (n = 22) in the Tat first exon region (Pattern Tat). As shown in table 2, in this case the recombination pattern also changed over time becoming more frequent the sequences presenting a recombination breakpoint in the Vpr.

**Table 2:** Distribution and frequency of recombination patterns in Vpr/Tat sequences

**Day**	**Pattern Vpr**	**Pattern Tat**	**Total**
**3**	6 (42.8%)	8 (57.2%)	14

**7**	9 (52.9%)	8 (47.1%)	17

**10**	11 (73.3%)	4 (26.7%)	15

**18**	13 (86.7%)	2 (13.3%)	15

The analysis of nucleotide positions involved in recombination showed that the most frequently affected ones were: 5757–59, 5766–68, 5788–90 (Vpr residues L67, I70 and R77, respectively), 5855–57, 5864–66, and 5876–78 (Tat residues E9, K12 and S16, respectively).

Vpr sequences encompassing aminoacids 55 to 96 (α-helix 3 and carboxi terminal domains) were studied. The α-helix 3 is thought to account for the formation of Vpr dimers and/or for the interaction with cellular partners, while the C terminal domain has been shown to participate in cell-cycle arrest, one of the most important function ascribed to Vpr.

Noteworthy, Q77R, a substitution not present in the parental strains used in this study, was found in the Vpr/Tat recombinant sequences. Moreover, its frequency was found to be on the increase as follows: in 14.3% of the sequences from day 3 (2/15), 23.5% on day 7 (4/17), 26.7% on day 10 (4/15) and 60% on day 18 (9/15). Sequences harboring Q77H substitution were not found. Frequency of this Q77R in naturally occurring subtype B, F and BF Vpr sequences was found to be 47.8% (n = 257), 11.8% (n = 17) and 26% (n = 50), respectively. All the analyzed sequences were obtained from Los Alamos Sequence Database.

No changes were observed in Vpr residues L67 and I70 in the analyzed sequences. As above mentioned, codons for theses residues were frequently affected by recombination events.

T84I substitution was also present in the recombinant sequences (30.9% of them) and always associated to the Q residue at position 77. This aminoacidic change is not present in the parental B or F1 strains used in this study and it has been found in approximately 50% of the naturally occurring BF recombinants.

Other important aminoacidic positions, A59, S79, R80, R90 S94 and S96 were analyzed. A59 has been shown to be involved in Vpr protein incorporation into virions [17,18], while S79, R80, R90, S94 and S96 have been directly related to the cell-cycle arrest function [19]. Phosphorylation of 79S, 94S and 96S has been suggested to regulate a putative nuclear localization signal (NLS) function [20], and changes in these positions may affect protein localization. Besides, it has been reported that substitutions in positions 80 and 90 impaired the G2-arrest and apoptotic activities without affecting the nuclear envelope localization [21].

One sequence from day 7 and 1 from day 10 (3.3%) harbored the A59T substitution, 2 from day 7 and 1 from day 10 (4.9%) harbored the S79N, 1 from day 10 (1.6%) harbored the S94V alone, and 9 (14.7%) from day 18 harbored both S94V and S96P, simultaneously. Our results also exhibited that all 9 sequences harboring S94V and S96P also harbored R77Q and T84I.

Regarding positions 80 and 90, none of the sequences presented changes in position R80, but R90K substitution was found in 1 sequence from day 7 (5.9%) and 2 sequences from day 10 (13.3%), and R90E substitution was found in 9 sequences from day 18 (60%).

On the other hand, the Tat N-terminal (residues 1–21), cystein-rich (residues 22–37), and core (residues 38–48), coding sequences were analyzed. These 3 domains are also known as Tat activation domain.

As regards the Tat N-terminal or acidic domain, it is predicted to form an α-helix. In addition to the negatively charged amino acids placed in this domain, B subtype Tat has 2 positively charged residues (R7 and K12) that are likely to stabilize the secondary structure, while F1 subtype has N in the above mentioned positions.

These 2 positions were analyzed together, i.e. R7-K12 or N7-N12. Our findings showed that R7-K12 and N7-N12 co-existed in samples from day 3, 7 and 10, with a mean frequency of 32% and 35%, respectively. Nevertheless, by day 18 N7-N12 was found in 80% of the sequences. Of the two possible combinations between these 2 patterns, R7-N12 and N7-K12, we only found the former in 12% of the sequences from day 7, 10 and in 13% of the sequences from day 18.

To note, at the end of our experiment (day 18), 85% of the sequences harbored the N7-N12 pattern. This association has also been frequently observed in naturally occurring BF recombinants Tat sequences [14].

Q17, a highly conserved residue in B and F1 Tat proteins, is frequently changed to K or R in natural BF recombinants (14.7%, n = 61) (sequences taken from Los Alamos Database). Q17K substitution was found in 13.3% of the sequences from day 18 only. N24K substitution, not present in the parental B or F but found in the prototypic BF CRF12_BF and other natural BF recombinants, was found in 2 sequences, one from day 7 and 1 from day 18. R29, another substitution typically observed in natural BF recombinants, was found in 41.8% of the sequences. This frequency increased over time, being 18% on day 3, 31% on day 7, 34% on day 10 and 78.5% on day 18.

K28, target for PCAF and p300 acetyltransferases and crucial for transcriptional activation, K41, essential for Tat activity [22,23], and the cystein residues present in the C rich domain, were found to be conserved in all the sequences.

### Relative fitness evaluation

**Figure 3:**
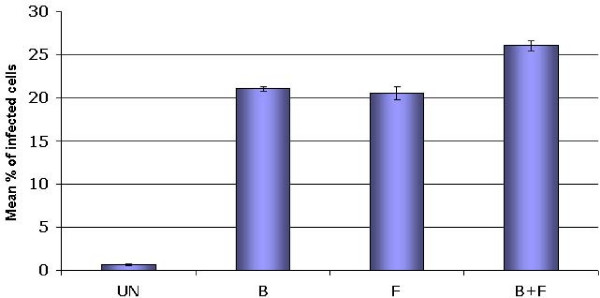
**Relative infectivity of mono (B or F Subtype) and dual-infected (B+F) cell culture viral populations**. Supernatant from mono an dual-infected cultures from day 18 were collected and used to infect GHOST indicator cell line. Viral inoculums were standardized by P24 antigen content. Forty eight hour post infection percentage of infected cells was measured by flow cytometry. The mean percentage of infected cells for each case are shown. Error bars indicate standard errors.

After completing the sequences analysis we aimed at determining if recombination had a measurable impact on viral population fitness, and infectivity was used as a representative value of it. Thus, mono (B and F) or dual (B+F) infected cell cultures supernatants were titrated by p24 antigen concentration and used to infect GHOST X4 cell cultures. At 48-h post-infection, percentage of GFP (+) cells was measured by FACS. Results showed a slight but significant (p < 0.05) increase in infectivity for the B+F supernatant from day 18 (26.5%) when compared with B (21.1%) and F (20.5%) (Figure 3).

## Discussion

Interactions between the cis-acting elements present in the viral promoter (LTR), the viral proteins and the transcription factors present in the host cell, influence the levels of viral gene expression under a wide variety of conditions. The LTR, although not absolutely necessary for viral replication, responds to activation signals that stimulate its activity increasing the rate of viral production.

In this report we present data that shows how the intersubtype recombination phenomenon impacts at the sequence level, on two different but functional related regions of the HIV-1 genome: LTR and Vpr-Tat. The results indicate that BF intersubtype recombination occurs in a space-time fashion, since this phenomenon took place first in the Vpr-Tat region and later on in the LTR-Gag region. Significant differences were found regarding recombination breakpoints, in terms of its number and distribution over the time. The number of breakpoints was observed to be higher in the former than in the latter, and breakpoints in the LTR showed a more restricted location pattern. The small number of breakpoints within the LTR could be ascribed to the fact that recombinants with breakpoints in this region might likely tend to be selected against.

Analysis of the major transcription factor binding sites present in the LTR revealed a high conservation of most of them. Nevertheless, a remarkable high frequency of sequences harboring mutations in the NFkβ and Sp1 binding sites was found. It has been recently demonstrated that NFkβ and Sp1 sites present in the HIV-1 LTR influence both, the gene expression levels and the dynamic switching between active or latent infection [24]. Mutations in NFkβ and Sp1 sites found in the analyzed recombinant sequences might have a significant impact on replication capacity of variants harboring them, since theses sequences seem to be positively selected over time.

Of the three Sp1 sites found in the U3 region of the viral promoter, mutations were highly frequent in site II, and to a lesser extent in site III. Mutations in these sites have been shown to be strongly correlated to alterations in viral replication [24,25]. The absence of sequences harboring mutations in the Sp1I site underscores its importance in viral gene regulation.

Regarding NFkβ, we found an increasing number of mutations affecting both sites over time. Site I was the first to show nucleotide changes, but mutations were soon detected in site II. It has been shown that functional roles of these two sites are not redundant, having site I an activating role and site II a repressing one. Thus, mutations affecting these sites in different time points could function as a delicate mechanism of regulation of viral gene expression by compensating any losses or gains in transcription levels. This mechanism may play a key role in the adaptation process of recombinant variants.

The observed structural variations on the Vpr-Tat coding region from recombinant sequences may be related to changes found in the LTR, since it is well documented that transactivation of HIV-1 gene expression is mainly induced by Tat through the interaction with TAR, and by the interaction of Vpr with cis-acting elements, including NF-κB, Sp1, C/EBP and the GRE enhancer sequences [16,26,27]. Vpr has also been shown to interact with the ubiquitous cellular transcription factor Sp1 [28] and that can directly bind to p300 via interaction the C-terminal α-helix 3 of the protein [29] suggesting that Vpr may act by recruiting them to the HIV-1 promoter thus enhancing viral expression.

On the other hand, the high frequency of the R77Q substitution found in the recombinant sequences highlights the relevance of this residue in the context of genomes evolution after intersubtype recombination events. Although controversial, some reports showed that R77Q is frequently found in viruses isolated from long term non-progressor HIV-1 infected patients. Experimental results revealed that viruses harboring this mutation have diminished their capacity to induce apoptosis, or on the contrary, the presence of such mutation would have a moderate positive effect on cell viability thus increasing the viral replication capacity [30-32]. As shown in results, sequences representing recombinant viral population at the end of the experiment also exhibited a high frequency of mutations in the Vpr residue R90 that has been shown to affect one of its more relevant functions, i.e. cell cycle control and apoptosis [21]. Altogether, this data suggest that rapid and significant changes in viral pathogenesis may result from intersubtype recombination affecting this accessory protein.

Despite the observed changes in Tat N-terminal (acidic, cystein rich and core regions) coding sequences have not been directly associated to specific protein functions, recombinant sequences analyzed here show alterations that have also been observed in naturally occurring BF recombinants, suggesting a relationship between recombination events and structural/functional modifications.

Finally, the evaluation of relative viral fitness revealed a slight but significant increase in infectivity of the viral population originated from the B+F dual infected cell culture at the end of the time course-experiment. Therefore, it can be hypothesized that after several rounds of recombination events and selection, this mixed population may become enriched in viral forms with higher replication capacity, underscoring the relationship between sequence evolution and fitness variations.

## Conclusion

Considering the stated above, one may speculate that the observed changes in the studied viral promoter and/or the Vpr-Tat sequence are a consequence of a mechanism that promotes biological adaptation and compensates any possible fitness loss after recombination involving these regions. Although the experimental model developed in this work only allowed to identify a limited number of recombinant forms, our results demonstrate that the study of recombination patterns evolution has the potential to provide insight into key dependencies between intra- and inter-viral genomic regions.

In summary, this work provides useful data on the consequences of HIV-1 intersubtype recombination, opening a window to better understand its role in generating novel strains and inducing viral fitness changes, which would have important implications on HIV-1 epidemiology and pathogenesis.

## Methods

### Cells and viruses

NL4-3 (GenBank: AF324493) and 93BR020 (GenBank: AF005494) strains were used for B and F subtypes respectively. Viral infections were carried out in MT2 cell cultures grown in RPMI 1640 medium (Cellgro; Mediatech, Herndon, VA) supplemented with 10% FBS, penicillin/streptomycin (100 U/ml, 100 mg/ml), L-glutamina (2 mM) and sodium bicarbonate. GHOST X4 cells, used to perform relative infectivity assay, were grown in DMEM medium (Cellgro; Mediatech, Herndon, VA) supplemented with 10% FBS, L-glutamine (2 mM), penicillin/streptomycin (100 U/ml, 100 mg/ml), G418 (300 ug/ml), hygromycin (100 ug/ml) and puromycin (1 ug/ml). Cell lines and viral strains were obtained through the AIDS Research and Reference Reagent Program .

### Viral Infections

For viral infections, 2 × 10^6 ^MT2 cells were infected with B or F and B+F viruses separately at a multiplicity of infection (MOI) of 0.01 IU/cell. B and F were mixed before cells addition, and then incubated for 2 hs at 37°C in a 5% CO2-containing atmosphere.

Viral infections were maintained up to 18 days post-infection by adding fresh cells when cytopathic effect was evident: 2 × 10^6 ^uninfected cells were mixed with 500 ul of double and single-infected cultures (cells plus supernatant). Sampling was performed at days 3, 7 and 10. Cell culture samples were centrifuged for 10 minutes at 2500 RPMs in order to separate cells from supernatants. Pelleted cells and aliquoted supernatants were stored at -80°C until use.

### PCR

Subtype-specific PCRs were designed to amplify the proviral genome regions under study: one comprising the viral promoter and part of the Gag coding sequence (positions 128 to 944 in the HXB2 numbering), and the other including the 3' half of Vpr and 5' half of Tat coding sequences (positions 5683 to 5973 in the HXB2 numbering). Primers for these reactions were designed based on parental B and F subtype sequences: LTR-Gag B fw 5'-G**C**A **A**GT AGA A**G**A GGC CAA T**A**-3', LTR-Gag B rev 5'-**G**TT **A**AT CC**T **GGC CTT **T**TA G-3', LTR-Gag F fw 5'-G**G**A **G**GT AGA A**A**A GGC CAA T**G**-3', LTR-Gag rev 5'-**C**TT **G**AT CC**A **GGC CTT **C**TA G-3', Vpr-Tat B fw 5'-TA GGA CAA CAT ATC TAT **G**A**A **AC**T**-3', Vpr-Tat B rev 5'-**A**A A**A**G **C**CT TAG GCA TCT CCT ATG-3', Vpr-Tat F fw 5'-TA GGA CAA CAT ATC TAT **A**A**C **AC**C**-3', Vpr-Tat F rev 5'-GA A**G**G **G**CT TAG GCA TCT CCT ATG-3'. Cycling conditions used were: 95°C for 5 min, followed by 35 cycles of 95°C 45 s, 60°C 45 s, 72°C 1 min for the LTR-Gag region, or 35 cycles of 95°C 45 s, 58°C 20 s, 72°C 1 min, for the Vpr-Tat region, and a final extension of 5 minute to 72°C.

Cellular DNA was quantified to control for the input of genetic material in each PCR reaction

pNL4-3 and p93BR020.1, as well as a plasmid containing the Vpr-Tat genomic region of CRF12_BF (ARMA159) obtained in a previous work (unpublished) were used as controls for the amplification reactions.

The two possible selected recombinant structures, B/F or F/B, were detected by using primer combinations: LTR-Gag B fw/LTR-Gag F rev or LTR-Gag F fw/LTR-Gag B rev, and Vpr-Tat B fw/Vpr-Tat F rev or Vpr-Tat F fw/Vpr-Tat B rev. The specificity of each primer combination was tested (Figure 1).

Intersubtype recombinant (from dual-infected cultures) as well as B and F (from mono-infected cultures) PCR products were gel-purified (QIAquick gel extraction kit, QIAGEN GmbH, Germany) and cloned into a commercial cloning vector (pGEM T Easy, Promega, USA).

Nucleotide sequencing was performed using a Big Dye Terminator sequencing kit (Amersham, Sweden) and an automatic sequencer (Applied Biosystems DNA sequencer 3100).

### Sequence analysis

A multiple alignment of the sequences with reference sequences was performed using Clustal W, and visually corrected with the BioEdit version 5.0.9 program .

Sequences were analyzed using a tool for the detection of intersubtype genomic recombination in HIV-1, available online at . This method uses a probabilistic approach to compare a sequence to a multiple alignment of a sequence family[33]. Recombination events were confirmed by bootscanning analysis as implemented in the SimPlot v.3.5.1 program .

LTR sequences were analyzed in search of transcription factors binding sites (TFBS) using a web-based method (TFSEARCH Data Base, available at ).

Mutations analysis was performed by visual inspection of the sequences.

B and F sequences from mono infected cultures were used as control for sequence stability over the time.

### Infectivity Assay

Relative infectivity assay was performed by infecting GHOST X4 cell cultures with mono (B and F) and dual (B+F) culture supernatans from sampling days 3, 7, 10 and 18, and the proportion of GFP+ cells was measured 48 hours post-infection using a FACScalibur flow cytometer (BD Biosciences, San Jose, CA). The percentage of GFP expression was normalized to the control uninfected cell culture. Culture supernatans were previously titrated by p24 antigen concentration through a commercial ELISA assay (Murex, Abbott, USA), including a calibration curve. All experiments were performed in duplicate.

### Statistical analysis

All data was expressed as mean ± SD. Significance (p < 0.05) between means of two experimental groups was evaluated using the Student's t test for independent samples (Primer of biostatistics 4.02 statistical program).

## Competing interests

The authors declare that they have no competing interests.

## Authors' contributions

MC and CCR contributed equally to this work designing and performing the experiments and during the manuscript preparation. CDC participated providing technical help during cloning and sequencing procedures. GT contribute to design the study and helped with phylogenetic analysis and data interpretation. HS supervised experimental design and writing of the manuscript. All authors read and approved the final manuscript.
